# Propagating Surface Plasmon Polaritons: Towards Applications for Remote‐Excitation Surface Catalytic Reactions

**DOI:** 10.1002/advs.201500215

**Published:** 2015-10-28

**Authors:** Zhenglong Zhang, Yurui Fang, Wenhui Wang, Li Chen, Mengtao Sun

**Affiliations:** ^1^ Beijing National Laboratory for Condensed Matter PhysicsInstitute of PhysicsChinese Academy of SciencesBeijing100190P.R. China; ^2^ Leibniz Institute of Photonic TechnologyAlbert‐Einstein‐Str. 907745JenaGermany; ^3^ Division of BionanophotonicsDepartment of Applied PhysicsChalmers University of TechnologyGothenburgSE‐412 96Sweden; ^4^ School of ScienceXi'an Jiaotong UniversityXi'an710049China; ^5^ Material Science and EngineeringUniversity of CaliforniaSan DiegoCA92093USA

**Keywords:** plasmonic waveguides, remote excitation, surface catalytic reactions, surface‐enhanced Raman scattering

## Abstract

Plasmonics is a well‐established field, exploiting the interaction of light and metals at the nanoscale; with the help of surface plasmon polaritons, remote‐excitation can also be observed by using silver or gold plasmonic waveguides. Recently, plasmonic catalysis was established as a new exciting platform for heterogeneous catalytic reactions. Recent reports present remote‐excitation surface catalytic reactions as a route to enhance the rate of chemical reactions, and offer a pathway to control surface catalytic reactions. In this review, we focus on recent advanced reports on silver plasmonic waveguide for remote‐excitation surface catalytic reactions. First, the synthesis methods and characterization techniques of sivelr nanowire plasmonic waveguides are summarized, and the properties and physical mechanisms of plasmonic waveguides are presented in detail. Then, the applications of plasmonic waveguides including remote excitation fluorescence and SERS are introduced, and we focus on the field of remote‐excitation surface catalytic reactions. Finally, forecasts are made for possible future applications for the remote‐excitation surface catalysis by plasmonic waveguides in living cells.

## 1 Introduction

Surface plasmons (SPs) are the collective oscillations of free electrons that are confined evanescently on a metal surface.^1^ Coupled with photons, SPs can act as a collective excitation of conduction electrons that propagate in a wave like manner along an interface between a metal and a dielectric, known as surface plasmon polaritons (SPPs). SPPs are confined to the vicinity of the interface and can propagate along the metal surface until the energy dissipates, either by heat loss or by radiation into free space.^2^ Localized surface plasmons (LSPs) are oscillations of charge density that are confined on the metallic nanoparticle's surface. The resonant LSPs, known as LSPR, result in a large enhancement of the localized electromagnetic (EM) field, which is the underlying mechanism for surface enhanced spectroscopy. LSPs and SPPs have been widely used in plasmonic resonance sensors,^2^, ^3^ tip‐/surface‐enhanced Raman spectroscopy, so‐called TERS/SERS,^4^, ^5^ and plasmonic catalysis,^6^ etc.

Since their discovery, SERS and TERS have extensively been experimentally and theoretically studied because of their extremely high sensitivity and wide applications in qualitative and quantitative analysis by fingerprint vibrational spectroscopy, even at the single‐molecular level.^7^, ^8^, ^9^, ^10^, ^11^, ^12^, ^13^, ^14^ In addition, due to the high throughput and low energy requirements, a novel application of plasmonics in chemical reactions gains significant attention, as reported in works on dissociation of hydrogen^15^, ^16^ and water,^17^, ^18^ photochemical^19^, ^20^, ^21^, ^22^, ^23^ and plasmon‐driven chemical reactions,^24^, ^25^, ^26^, ^27^, ^28^, ^29^, ^30^, ^31^, ^32^, ^33^, ^34^, ^35^, ^36^, ^37^ etc. SPs excited on the surface of gold or silver can non‐radiatively decay into so‐called hot electrons with a high energy between the Fermi and vacuum energy level.^36^, ^37^, ^38^, ^39^, ^40^ The hot electrons could scatter into the absorbed molecule's excited state and then trigger chemical reactions by reducing the activation energy, so‐called plasmonic catalysis.^41^ If the reaction is monitored by SERS or TERS spectra, the nanostructures behave as catalysts and nanoscale signal enhancers, simultaneously. Plasmon catalyzed reactions monitored by TERS and SERS have been reviewed by many different groups.^5^, ^6^, ^42^, ^43^, ^44^, ^45^, ^46^, ^47^

**Figure 1:**
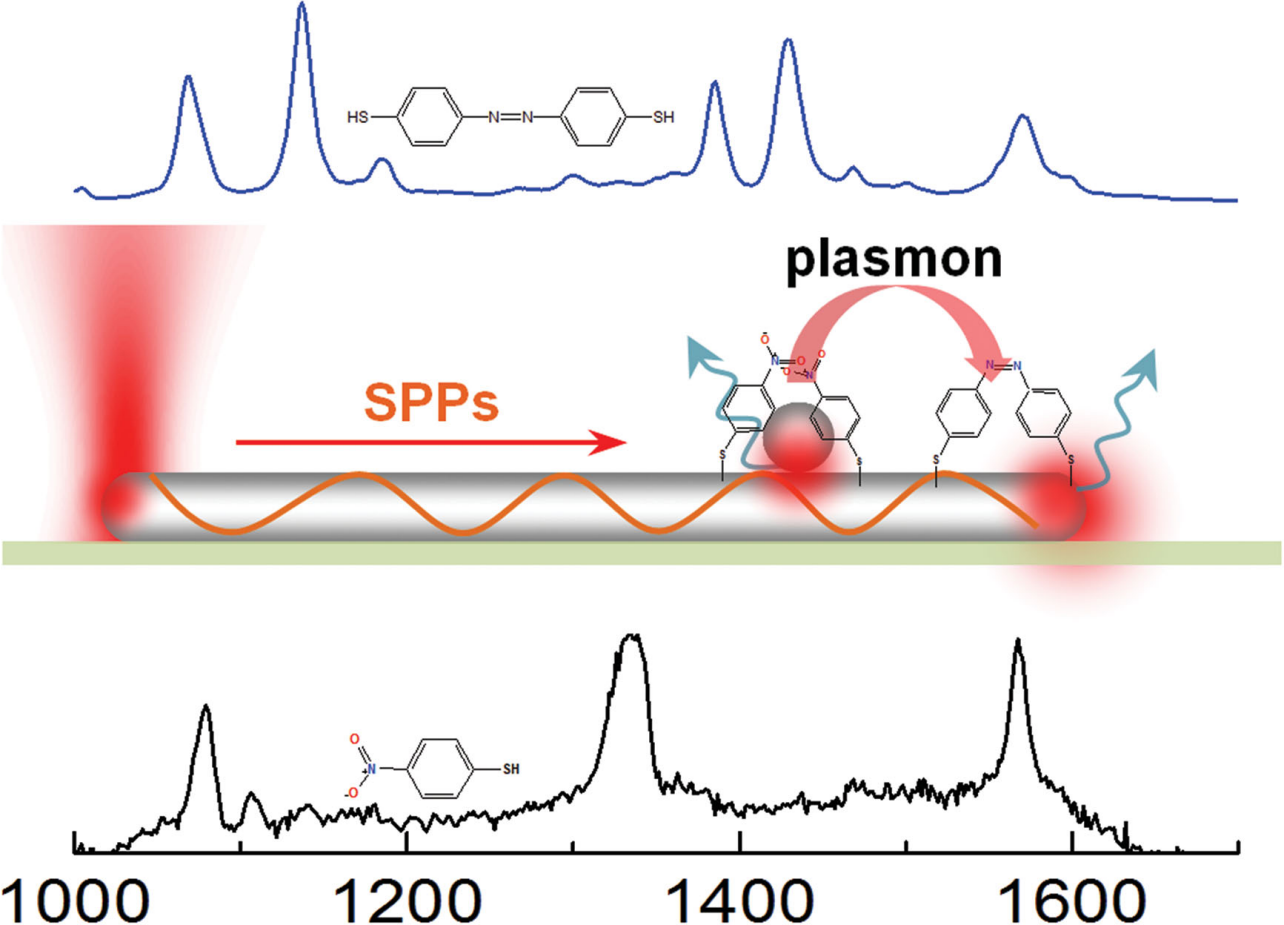
Sketch of remote SERS based on a silver nanoparticle–nanowire system. Raman signals obtained from the excited side is local SERS, while the signals collected at the junction where energy comes from propagating SPPs is remote SERS.

Currently, the plasmonic waveguide has been successfully applied in the field of remote‐excitation surface enhanced Raman spectroscopy.^48^, ^49^ Compared with traditional SERS, remote excitation SERS has many advantages. In normal SERS, the excited light is focused on the detected spot, which is called local SERS. In contrast in the ‘remote SERS’, the excited spot is far away from the detected target, which is excited by the SPPs with a ‘remote mode’. Ag or Au waveguides offer a way to go below the size limit because they transfers optical signals via SPPs. SPPs can propagate along the metal waveguide and emit free photons at imperfections or at the end of waveguide, while some of them are lost due to the Ohmic damping. The energy transfer from the spot of illumination and the hot‐spot can be mainly supported by the remote systems based on plasmonic waveguide, as illustrated in **Figure** **1**. The SPPs could of course excite the nanoscale hot‐spot remotely, which can avoid the strong background noise because of a large area of the excited spot, and isolate heat effects from the area of excited light to the target excited in the area of sub‐diffraction wavelength. Moreover, it can reduce the possibility of the sample damage by high laser excitation. Based on surface‐enhanced spectroscopy, a novel way of sensing measurements can be provided by such a remote SERS system, and it can be used in systems where normal SERS is unsuitable, such as Raman and fluorescence detection of biomolecules in living cells.^50^, ^51^, ^52^

It is important that the plasmon‐catalyzed reactions can be remotely driven by silver plasmonic waveguides.^53^, ^54^, ^55^ In the plasmon‐driven chemical reactions via the traditional SERS method, the laser is directly radiated on the molecules and metal substrate. Several environmental factors for the plasmon‐driven chemical reactions are difficult to clearly exclude, such as photons generated directly from light of the laser, electrons directly escaping from the metal surface by overcoming the work function (photoelectric effect), and heating effect of the laser or surface plasmonic resonance. The plasmonic‐waveguide‐driven surface catalytic reactions can provide more confident evidence for the mechanism and nature, since the above several environment factors can be explicitly excluded.

In this review, we introduce the recent progress of the propagating SPP (plasmonic waveguide) driven surface catalytic reaction. First, we introduce the synthesis and characterization of plasmonic waveguides of Ag nanowires (NW), and the propagating mechanism of the Ag nanowire is physically revealed. Then, we focus on the application of plasmonic waveguide for remote‐excitation research, including fluorescence and SRES, and surface catalyzed reaction of *pp*′‐dimercaptoazobenzene (DMAB), as revealed by remote excitation SERS spectroscopy. DMAB can be produced from *para*‐aminothiophenol (PATP) and 4‐nitrobenzenethiol (4NBT) by propagating SPPs; plasmonic waveguide‐driven reduced and oxidized reactions, respectively. Finally, we provide an outlook for possible future applications on remote‐excitation surface catalytic reactions in living cells.

## 2 Synthesis of Ag Nanowire Waveguides

Detection of plasmonic waveguide for remote‐excitation surface catalytic reactions requires smooth surfaces, controllable size, and high crystallinity, as these factors decrease dissipative energy loss. SPPs in the visible spectrum can propagate with the least energy loss over tens of micrometers on silver nanowire. Lots of methods have been reported on the synthesis of sliver nanowires such as solution‐phase, vapor‐phase,^56^, ^57^ and microfabrication techniques.^58^ Ag NWs prepared by lithography methods, however, have the disadvantages of producing polycrystalline structures that have inevitable defects due to the metal evaporation process. These disadvantages can significantly shorten the propagation distance of SPP in NW waveguides by increasing scattering loss.^58^ On the other hand, controlling the diameter and morphology of Ag NWs through the vapor‐phase method is difficult.

**Figure 2:**
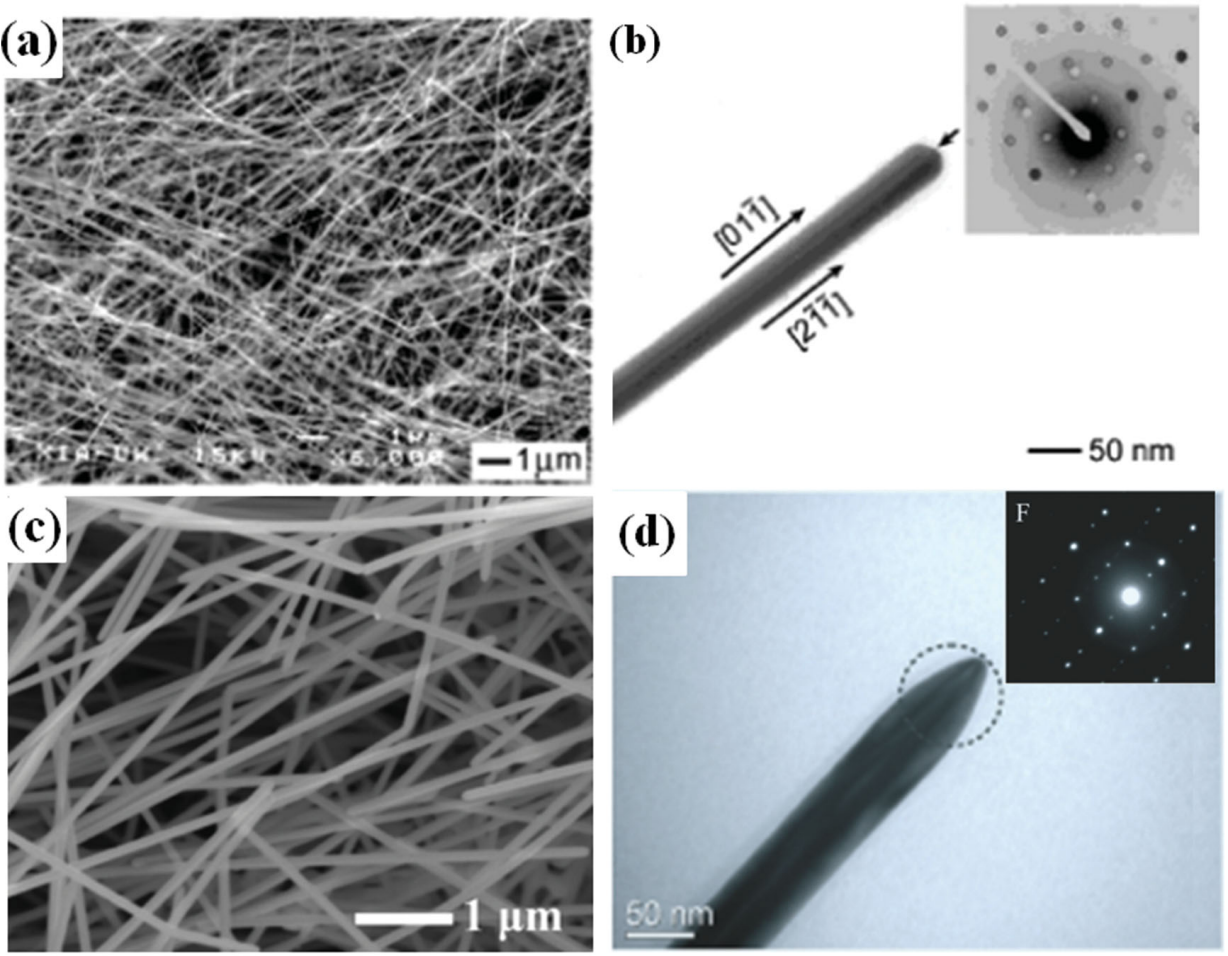
Chemical synthesized Ag NWs. a) SEM images of Ag NWs prepared through the conventional polyol synthesis process. b) TEM image of a Ag NW, demonstrating the bicrystallinity and corresponding growth directions. The inset shows the micro‐diffraction pattern recorded by focusing the electron beam onto this NW. c) SEM image of Ag NWs obtained via ammonium carbonate‐mediated polyol synthesis method. d) TEM image of an individual NW and the inset shows the selected‐area electron diffraction (SAED) patterns taken from the part indicated by the dotted circles. a,b) Reproduced with permission.^70^ Copyright 2002, American Chemical Society. c) Reproduced with permission.^71^ Copyright 2002, American Association for the Advancement of Science. d) Reproduced with permission.^82^ Copyright 2014, Elsevier.

**Figure 3:**
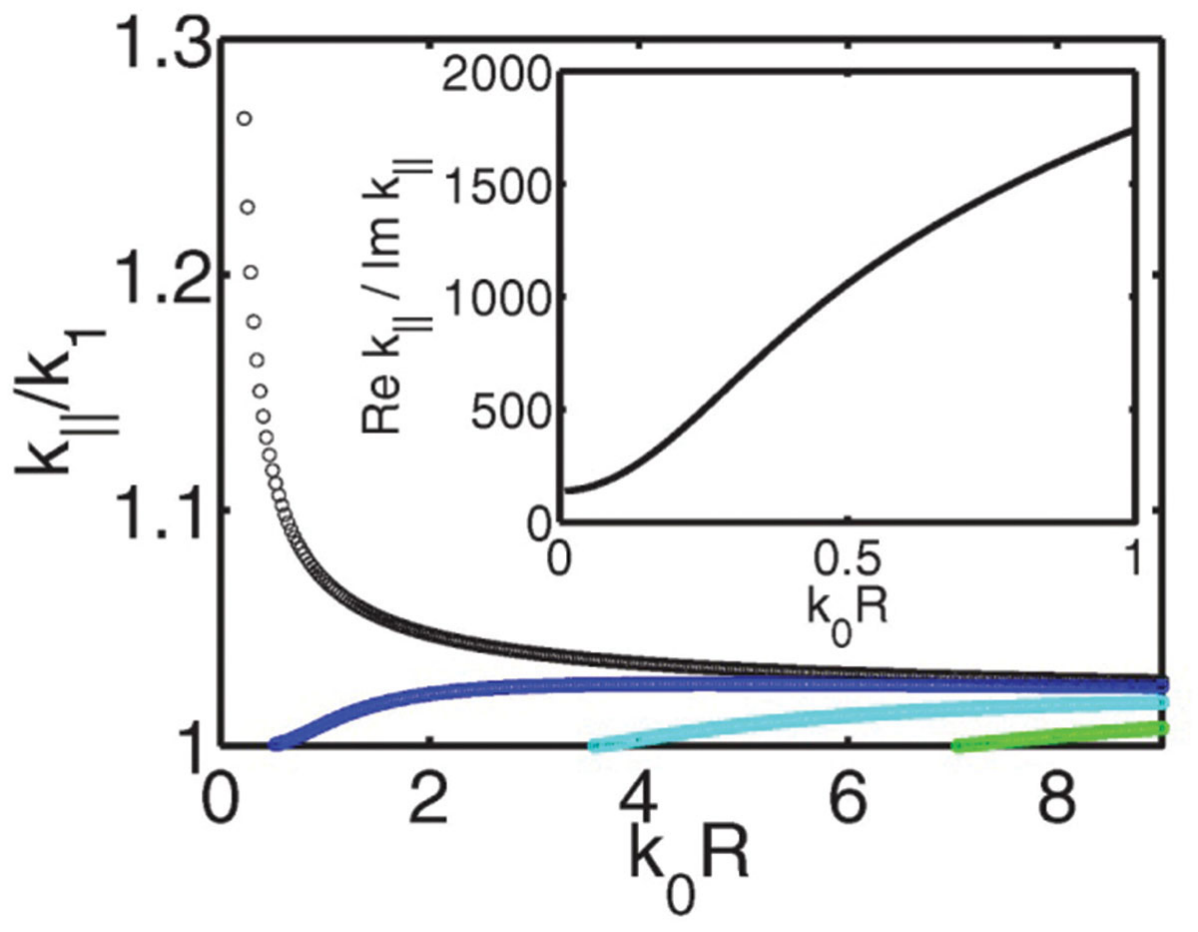
Allowed surface plasmon modes as a function of Ag NW radius (R). The surrounding dielectric *ε*_1_ = 2 corresponding wavelength λ_0_ = 1 μm. The *m* = 0, 1, 2, 3 are shown in black, blue, light blue, green. Reproduced with permission.^83^ Copyright 2007, American Physical Society.

Solution‐phase methods are divided into template and nontemplate methods.^59^, ^60^ The advantage of the template method is that it produces NWs with the desired morphology and high aspect ratio. Various one‐dimensional structures such as carbon nanotubes,^61^ mesoporous silica,^62^ alumina or polymer membranes,^63^, ^64^, ^65^, ^66^ DNA chains,^67^ and rodlike micelles^67^ are used as templates. However, it is too complex, and the shape and structure of the synthesized NWs are strongly dependent on the use of corrosive chemicals during template dissolution. These disadvantages may be avoided by adopting nontemplate solution‐phase methods.^68^ Nontemplate methods include the solution‐phase polyol process, which was developed by Xia et al.^69^, ^70^ It is widely used to prepare Ag nanowires with uniform morphology and size (**Figure** **2**).^71^ Silver nitrate in this process is reduced by ethylene glycol with vinyl pyrrolidone (PVP). Here, ethylene glycol is used not only as a solvent but also as a reducing agent, and PVP is used as a surfactant.^69^ The obtained silver atoms deposit onto seeds and grow into NWs with the aid of PVP. The reaction occurs at 160 °C. Agents such as NaCl, CuCl_2_, FeCl_3_, and PtCl_2_ may be added to the solution to ensure nanowire formation.^70^, ^72^, ^73^, ^74^ In this approach, the size of NWs is well‐controlled to within tens to hundreds of nanometers; their length can reach 100 μm. The morphology and size of NWs can be very precisely controlled by manipulation of experimental parameters such as reaction time, temperature, chemical ratio, injection speed, stirring speed, and added agents.^75^, ^76^, ^77^, ^78^, ^79^, ^80^, ^81^, ^82^ Furthermore, the structure of synthesized Ag NWs is highly crystalline, and the surfaces are very smooth. Dissipative energy loss can be drastically suppressed, and NWs can thereby be efficiently used in plasmonic waveguides for remote‐excitation surface catalytic reactions (**Figure** **3**).

## 3 Characterization of SPPs Propagating on Ag NW Waveguides

### 3.1 Basic Modes of SPP in Metal Cylindrical NW

This review is mainly concerned with SPP propagation in Ag NW waveguide, and most of the previous works consider a cylindrical shape of NW. Herein, we first describe SPP modes in an infinite cylinder. For an infinite cylindrical wire with radius *R* and permittivity ε_2_ in a homogeneous medium (ε_1_, a nonmagnetic material), Chang et al. describe the electric and magnetic field as follows:^83^

(1) $$E_{j}\left(r,t\right)=\left\{\left[\frac{im}{k_{j}\rho }a_{j}F_{j,m}\left(k_{j⊥}\rho \right)+\frac{ik_{∥}k_{j⊥}}{k_{j}^{2}}b_{j}F_{j,m}^{\prime }\left(k_{j⊥}\rho \right)\right]\right.+\left[-\frac{k_{j⊥}}{k_{j}^{}}a_{j}F_{j,m}^{\prime }\left(k_{j⊥}\rho \right)-\frac{mk_{∥}}{k_{j}^{2}\rho }b_{j}F_{j,m}\left(k_{j⊥}\rho \right)\right]\hat{\phi }\left.+\frac{k_{j⊥}^{2}}{k_{j}^{2}}b_{j}F_{j,m}\left(k_{j⊥}\rho \right)\hat{z}\right\}e^{i(m\phi +k_{∥}z-\omega t)}$$

(2) $$H_{j}\left(r,t\right)\;=\;-\frac{i}{\omega \mu_{0}}k_{j}\left\{\left[\;\frac{ik_{∥}k_{j⊥}}{k_{j}^{2}}a_{j}F_{j,m}^{\prime }\left(k_{j⊥}\rho \right)\;+\;\frac{im}{k_{j}\rho }b_{j}F_{j,m}\left(k_{j⊥}\rho \right)\;\right]\right.\hat{\rho }+\left[-\frac{mk_{∥}}{k_{j}^{2}\rho }a_{j}F_{j,m}\left(k_{j⊥}\rho \right)-\frac{k_{j⊥}}{k_{j}^{∥}}b_{j}F_{j,m}^{\prime }\left(k_{j⊥}\rho \right)\right]\hat{\phi }\left.+\frac{k_{j⊥}^{2}}{k_{j}^{2}}a_{j}F_{j,m}\left(k_{j⊥}\rho \right)\hat{z}\right\}e^{i(m\phi +k_{∥}z-\omega t)}$$

where *j* = 1, 2, a condition that pertains to the medium and wire. *F*_1,*m*_(*x*) = *H_m_*(*x*) and *F*_2,m_(*x*) = *J_m_*(*x*) are the Hankel and Bessel functions, respectively. $k_{2}^{2}=k_{j⊥}^{2}+k_{j∥}^{2}$ and $k_{j}^{2}=\frac{\omega }{c}\sqrt{\varepsilon_{j}}$. *a_j_*, *b_j_* are coefficients that are determined by boundary conditions. From the boundary condition, four equations can be obtained under the following condition:

(3) $$M{\left(a_{1}a_{2}b_{1}b_{2}\right)}^{T}=0$$

A solution only exists when

(4) detM=0

According to the results of Chang et al., only lower‐order modes (*m* = 0, ±1) could exist for thin metal wires; such modes are commonly used (Figure 3), and higher modes are thus excluded. The mode having which *m* = 0 always exists; the thinner the wire is, the more confined the electromagnetic field. For the mode in which *m* = ±1, there is no strict cutoff radius, but for a very small radius, the mode volume is very large. For the normal wires we used, modes for which *m* = 0 and *m* = ±1 exist. For very thick wires (*R* > 150 nm in air), higher modes exist. For the cases discussed in this paper, only normal wires (*R* ≈ 100 nm) are considered.

### 3.2 SPP modes of Ag NW by One End Excitation

In order to excite SPP in the NW, the simplest and most convenient way is directly focusing the light on one end of wire, due to the fact that local symmetry‐breaking at a finite length NW terminus is sufficient to excite SPP. Assuming that the harmonic monochromatic laser beam with electric field $E_{\mathrm{inc}}\left(r\right)=E_{0}\left(r\right)\cdot e^{-i\omega t}$ illuminate on the end of wire perpendicular to the wire, the modes on the wire will be excited with the following form (ignoring the time factor):

(5) $$E^{j}\left(r\right)=\sum_{m}{\hat{A}}_{m}^{j}F_{m}^{j}\left(k_{m⊥}^{j}\rho \right)\cos\;m\phi \cdot e^{ikm||z}$$

*m* = 0, ±1 corresponding to the modes which can be excited. For an ideal cylinder, in the electrostatic approximation, when the polarization is along the wire, m = 0 mode will be excited as

(6) $$E^{j}\left(r\right)={\hat{A}}_{0}^{j}F_{0}^{j}\left(k_{0⊥}^{j}\rho \right)e^{ik0||z}$$

If the polarization perpendicular to the wire, *m* = ±1 modes will be excited. According to the boundary condition, these are

(7) $$E_{1}^{j}\left(r\right)={\hat{A}}_{1}^{j}F_{1}^{j}\left(k_{1⊥}^{j}\rho \right)\cos\;m\phi \cdot e^{ik1||z}$$

Where *j* = 1, 2 refer to the medium around and wire. $F_{m}^{1}(x)=H_{m}(x),F_{m}^{2}(x)=J_{m}(x)$ are the Hankel and Bessel functions, respectively. ${\hat{A}}_{m}^{j}=a_{m}^{j}\hat{\rho }+b_{m}^{j}\hat{\varphi }+c_{m}^{j}\hat{z}$ is the vector coefficient.

**Figure 4:**
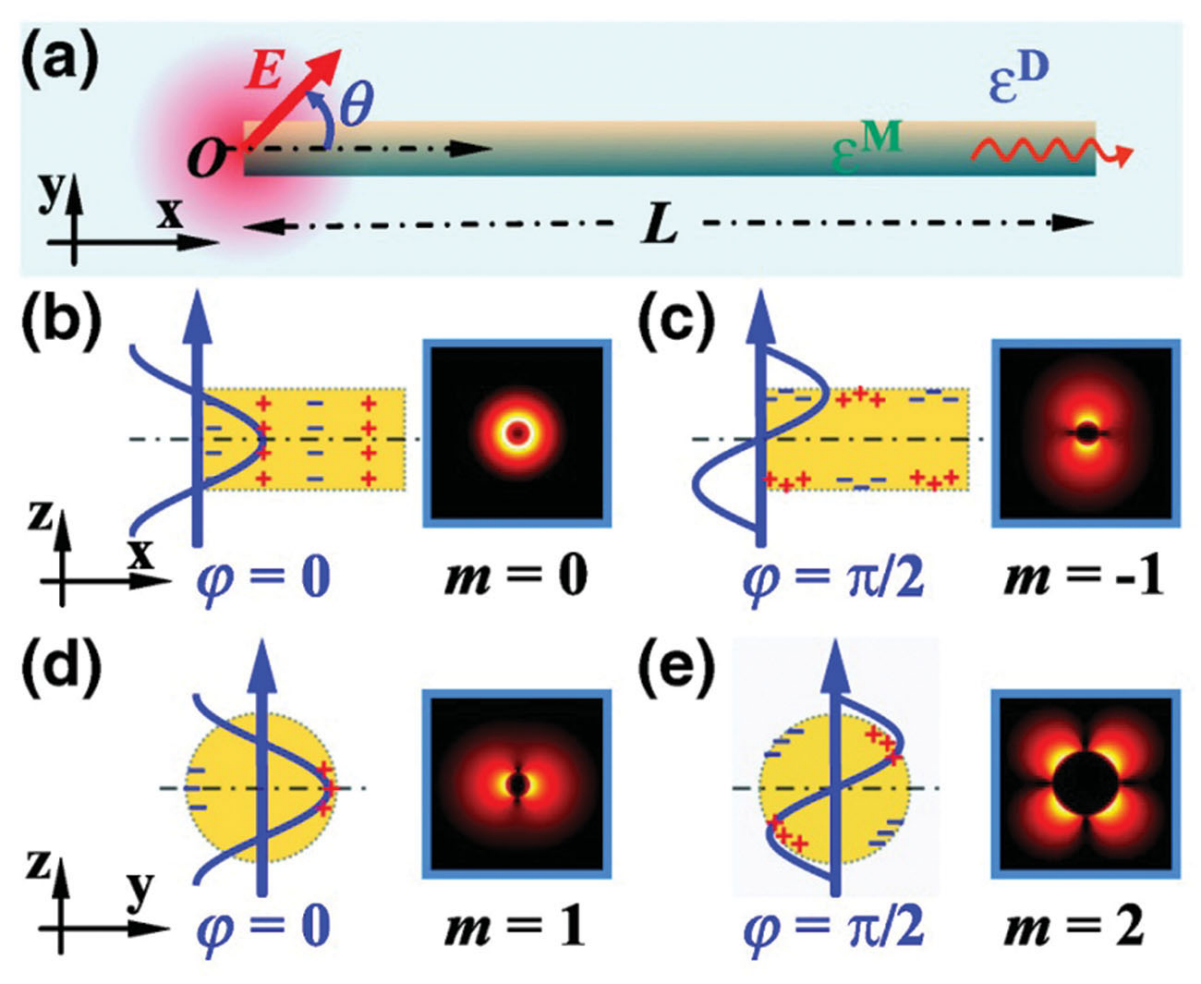
a) Schematic of excitation of plasmon modes in metallic NW in homogeneous environment. b–e) Plasmon modes excited at different incident polarization angle *θ* and incident phase *ϕ*. Reproduced with permission.^84^ Copyright 2011, American Physical Society.

As **Figure** **4** shows, the wire excited by a paraxial Gaussian beam with an instantaneous electric field of the form $E_{\mathrm{inc}}=E_{0}e^{-i\phi }$, where *ϕ* is the phase of incident light.^84^ The fundamental *m* = 0 SPP mode or *m* = –1 is selectively excited when the input light phase is *ϕ* = 0 or *ϕ* = π/2 with the polarization component along the wire. The *m* = 1 mode is generated for 00 *ϕ* = 0 with polarization components perpendicular to the wire. For any polarization, the light can be divided into the two basic components with polarization along or perpendicular to the wire. The amplitude of the components excited is strongly depending on the polarization angle and of course, the shape of the illuminating terminal.

## 4 Plasmonic Waveguide for Remote‐Excitation

### 4.1 Features of Remote‐Excitation SERS and Early Applications

**Figure 5:**
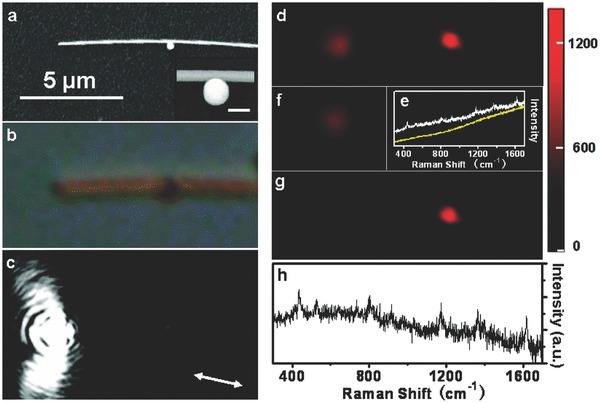
Remote‐excitation SERS of MGITC molecules excited through propagating plasmons. a) SEM, b) optical and c) SPP images of a nanowire−nanoparticle system. The corresponding Raman d) image and e) spectra of the Raman peak at 436 cm^−1^ excited at the left end of the nanowire. f) The fluorescence background image of smooth ITO glass only. g) The Raman image after background subtraction of panel (d) from panel (f). h) The remote‐excitation SERS spectrum after fluorescence background correction with the spectra taken from panel (e). Reproduced with permission.^49^ Copyright 2009, American Chemical Society.

SPPs propagating on noble‐metal nanowires have been widely studied in this decade, which sought to find the basic physical mechanism.^58^, ^83^, ^84^, ^85^, ^86^, ^87^ In 2009, remote SERS on single silver nanowire systems was investigated by Fang et al.^49^ Remote excited SERS of MGITC molecules was reported, alongside the polarization dependence on the single Ag nanowire with a nanoparticle that was excited by 632.8 nm wavelength (**Figure** **5**). The corresponding SEM, optical and SPP images are shown in Figure 5a–c. Normal SERS spectra of MGITC were obtained from the excited laser spot at left‐end of the Ag nanowire or the remote site of the gap of nanowire and nanoparticle, respectively. The remote SERS spectra were obtained from the remote site by separating the signal collection spot from the laser spot. Clearly, the remote excitation SERS spectra can be observed by a single Ag nanowire waveguide after the correction of fluorescence background. It was explained by a two‐step mechanism in the numerical simulation. First, the SPs are excited and propagate to the target spot; there is an electromagnetic field intensity enhancement (F_1_) for the excited wavelength. Then the intensity enhancement (F_2_) for the local structure of the target spot is achieved for the specific Raman peak wavelength. The product of the two factors gives the remote‐excitation SP signal enhancement. This mechanism works for the other systems in this review, while for the remote‐excitation catalysis, the first factor (for the laser) dominates, as the hot electrons are excited by the laser excited SPPs; the second factor mainly contributes to the Raman measurement of the catalyzed products. The Raman signal is also remotely detectable as part of the energy is converted to SPPs again as well.

**Figure 6:**
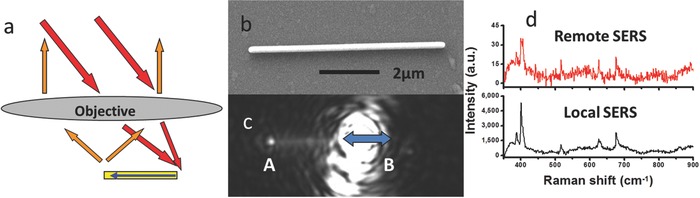
a) Sketch of remote SERS based on a single gold nanowire. b) SEM image and c) the corresponding SPPs image of the gold nanowire waveguide. d) The top remote SERS spectrum (red line) collected at terminus A and the bottom local SERS spectrum (black line) obtained at terminus B. Reproduced with permission.^87^ Copyright 2011, American Chemical Society.

Remote SERS was also measured by a single Au nanowire (**Figure** **6**).^87^ It was found that the remote excitation SERS is dependent on the polarization of the laser. Maximal Raman signals were obtained when the incident polarization is parallel to the nanowire waveguide. In this remote system based on a single nanowire waveguide, the polarization of the remote site could be set toward a specific direction by adjusting the polarization of the excited laser. This procedure, however, requires a previous measurement. Because of their antioxidant property, working time for the operation of Au plasmonic devices may be much longer under ambient environments compared with that of Ag plasmonic devices.

**Figure 7:**
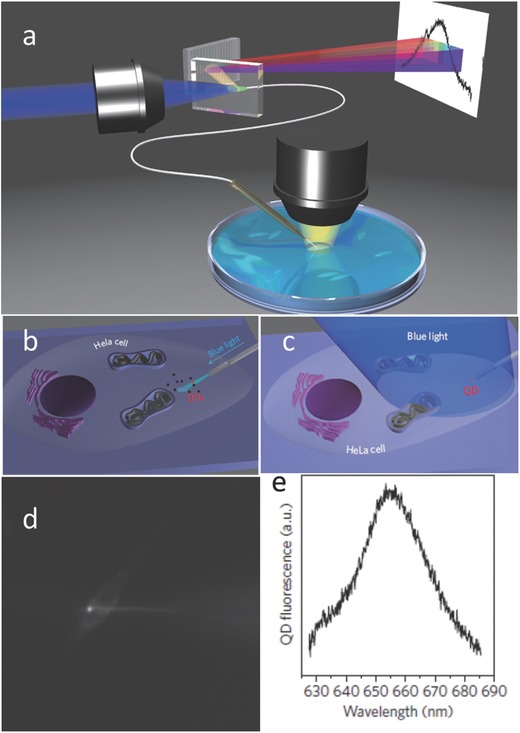
a) A sketch of the remote nanowire endoscope system in vivo cells. b) A sketch of sub‐cellular imaging and c) local spectrum remotely collected in a cell in vivo through the system. d) The dark field image of the remote detection of quantum dot fluorescence in a cell in vivo. e) The corresponding spectrum remotely collected through the nanowire end scope system. Reproduced with permission.^50^ Copyright 2012, Nature Publishing Group.^50^

The plasmonic waveguide for remote excitation has been tentatively used in the field of biology, chemistry and physics. In 2012, Yang's group reported a remote system based on nanowire for endoscopyin a cell, in vivo.^50^ The remote endoscopy consists of single nanowire of SnO_2_ attached to an optical fiber (**Figure** **7**) by using a three‐axis micro manipulating system. The inserted SnO_2_ nanowire could work as an optical waveguide supporter at the designated positions in vivo cells. The sub‐cellular images and the fluorescence of the quantum dots emitted at 655 nm in single cell were presented by using nanowire endoscope. In sub‐cellular spectra detection in remote endoscope system, the SnO_2_ nanowire was located around the preloaded quantum dots, which can be excited by 442 nm wavelength laser. As shown in Figure 7e, the fluorescence signals of quantum dots in cell could be then collected by the remote nanowire waveguide connected with a spectrometer and CCD. To achieve a larger intensity of the remote signals, a Ag or Au nanowire waveguide can be used to replace the semiconductor nanowire because of the enhanced electromagnetic of SPPs.

**Figure 8:**
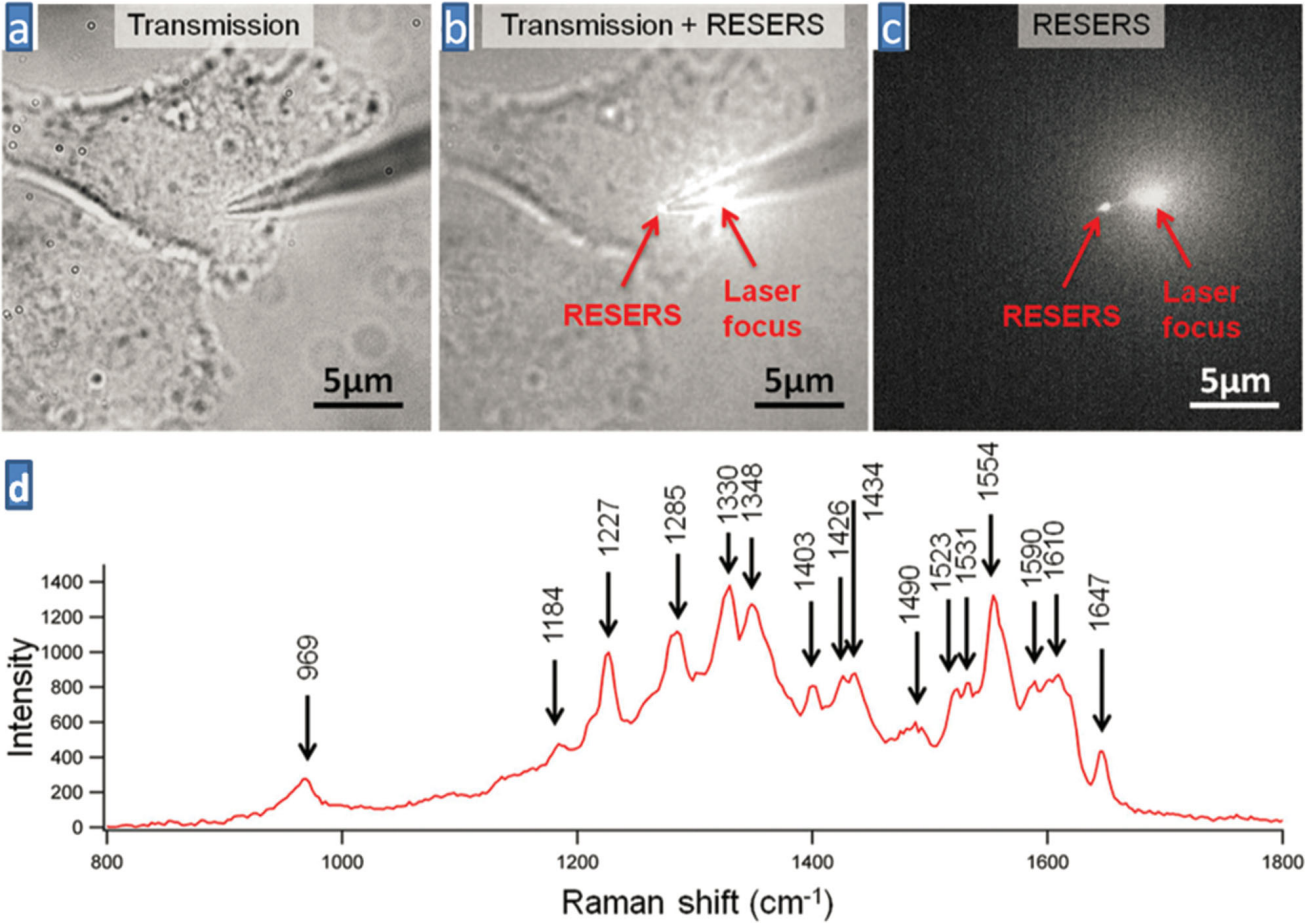
Scheme of the positions chosen for remote‐excitation SERS spectroscopy inside a live HeLa cell during Ag NW probe endoscopy. a–c), Remote excitation SERS endoscopy of a live HeLa cell. a) Optical transmission, b) combination of optical transmission and remote excitation SERS, and c) remote excitation SERS only images of a Ag NW probe in a live HeLa cell. d) A remote excitation SERS spectrum from the nucleus of the live HeLa cell. Reproduced with permission.^51^

In 2014, Uji‐i etal. reported the remote excitation SERS by plasmonic waveguide in living cell.^51^ As shown in **Figure** **8**, the plasmonic hotspot (at the gap of two Ag nanowires, or at the gap of nanowire and nanoparticles) on the Ag nanowire probe inserted into the cells was excited by SPPs, and the signals were collected from the other side the Ag nanowire probe outside the cells. A clear remote SERS spectrum can be observed by using an Ag nanowire probe hotspot inserted into a living HeLa cell.

Clearly, it is possible that plasmonic waveguide of a single metal nanowire can be used for remote excitation in living cell system. In addition, this remote excitation technique can also be used for another important application known as plasmon driven chemical reactions.

### 4.2 Remote‐Excitation Plasmon‐Driven Chemical Reaction

#### 4.2.1 Features of the Plasmon‐Driven Chemical Reaction

Assisted by the plasmon‐induced hot electrons, plasmonic catalysis was recently established as a very new heterogeneous catalytic reaction platform.^6^, ^24^, ^25^, ^32^, ^33^, ^37^, ^88^, ^89^, ^90^, ^91^, ^92^ Recent experimental and theoretical reports present plasmonic catalysis as a route to concentrate and direct the energy of visible light to adsorbed molecules, enhance the rate of chemical reactions, and offer a pathway to control the reaction selectivity. SPs can decay and transfer energy identical to that of incident photons to hot‐electrons with kinetic energies above Fermi level. Stronger laser intensity produces more surface plasmon quantum's, subsequently producing a large density of hot‐electrons generated from the plasmon decay. The hot‐electrons can jump into the unoccupied resonant molecular energy level on the surface of Ag/Au metal. As the hot electrons jump on the molecules, the molecular potential energy surface (PES) can be changed from a state of neutral to temporal negative‐ion. Energy from hot electrons above the Fermi surface can transfer intramolecular vibrational energy during the interactions, in which case the energy barrier of the chemical reactions decreases. In some cases,^29^ hot electrons provide kinetic energy and result in a temporal negative‐ion PES. But in some cases,^37^ additional electrons are also needed in the surface catalysis reaction. Therefore, hot electrons provide two or three important contributions to plasmonic catalysis.

**Figure 9:**
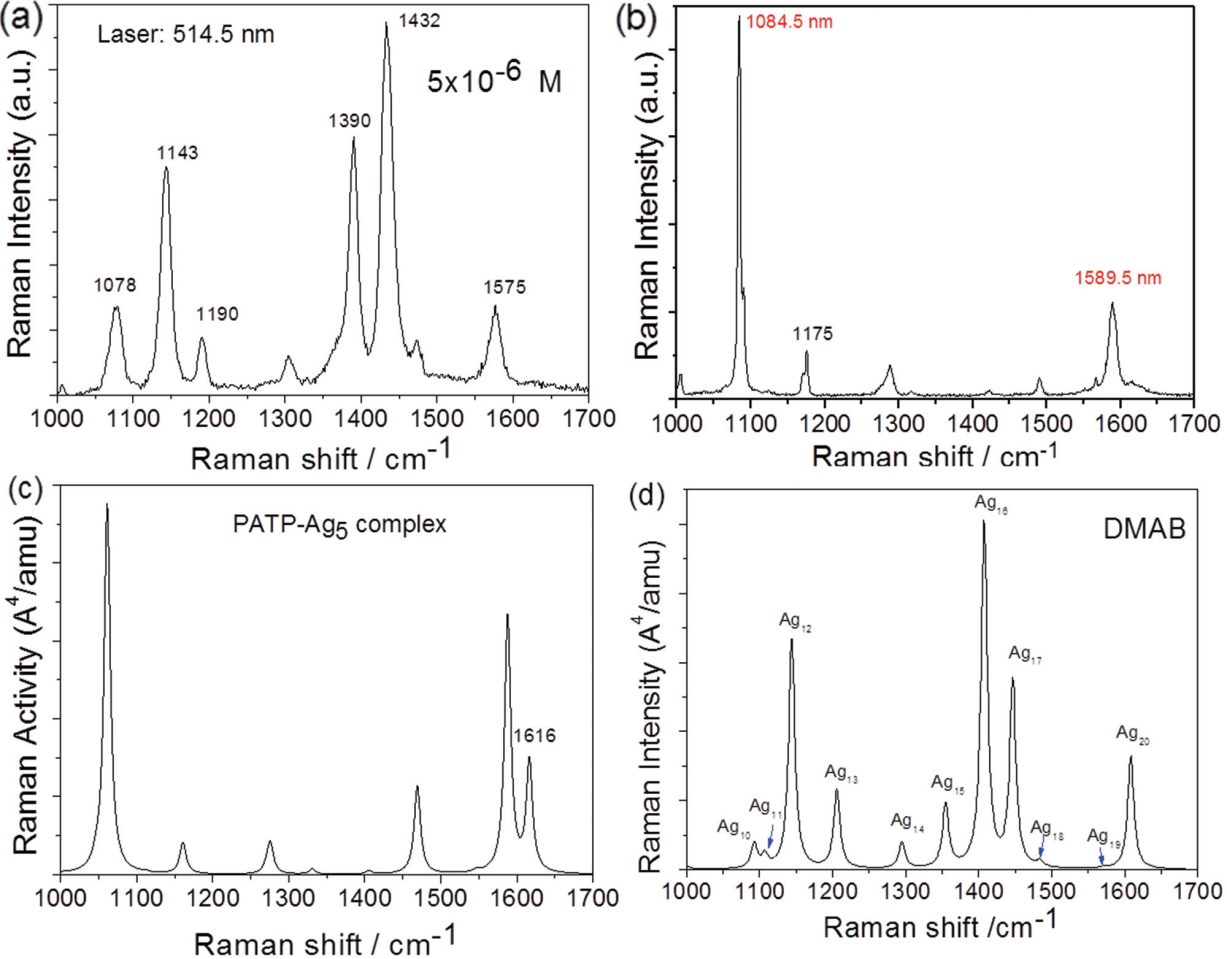
a) Experimental SERS spectra of DMAB excited at 514.5 nm, b) the experimental normal Raman spectrum of PATP, c) theoretical SERS spectrum of a PATP‐Ag_5_ complex, and d) simulated Raman spectrum of DMAB. Reproduced with permission.^24^ Copyright 2010, American Chemical Society.

A study in 2010 revealed convincing evidence of plasmon catalysis of PATP to DMAB by LSPs.^24^ As shown in **Figure** **9**, compare with the SERS and normal Raman spectra of PATP, it was found that additional Raman peaks are at 1143, 1390, and 1432 cm^−1^. However, simulated SERS spectra of PATP on Ag_5_ clusters could not be reproduced very well. Unexpectedly, the Raman spectrum of DMAB (Figure 9d) is well reproduced by SERS spectra of PATP in Ag sol.^24^ All the three strongly enhanced peaks of DMAB represent symmetric a_g_ vibrational modes, strongly indicating the formation of DMAB from PATP through the N = N bond. These peaks are assigned to the a_g12_, a_g16_, and a_g17_ symmetric vibrational modes, respectively.

**Figure 10:**
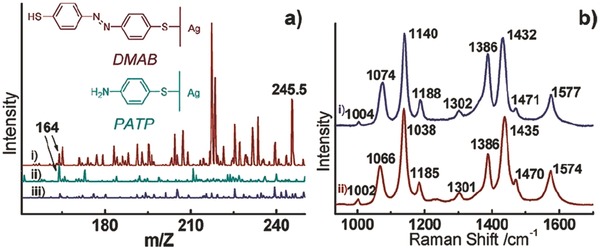
a) DESI (desorption electrospray inonization)‐MS spectra of PATP adsorbed on roughened Ag electrodes, illuminated with laser light i), without any irradiation ii), and a roughened Ag‐free spectrum of PATP iii). b) i) Raman spectrum of PATP, and ii) DMAB on the roughened Ag electrode excited by laser light at a power density of ca. 1 × 10^8^ mW cm^−2^. Reproduced with permission.^25^ Copyright 2010, American Chemical Society.

Meanwhile, Tian's group also found the same conclusion with more confident evidence, which revealed by SERS spectra and desorption electro‐spectra ionization mass spectrometry spectra.^25^ The plasmon assisted reactions of PATP to DMAB were experimentally well confirmed by surface mass spectrometry (SMS), and electrochemistry, and it was found that the results are well consistent with the calculations. The SMS spectra were obtained from the PATP‐adsorbed silver electrode after illuminated using a high power laser.^25^ As shown in **Figure** **10**, the peaks of SMS spectra at 245.5 and 164 m z^−1^ could be assigned to “H+S–Ph–N = N–Ph–S” fragment and “K+S–Ph–NH_2_”, respectively. The peak of 245.5 m z^−1^ in Figure 10a working as a control cannot be observed. It was found that the formation of DMAB was produced from PATP excited by a high power laser. Moreover, the SERS spectra also indicate that DMAB can be synthesized successfully, which is commercially unavailable

**Figure 11:**
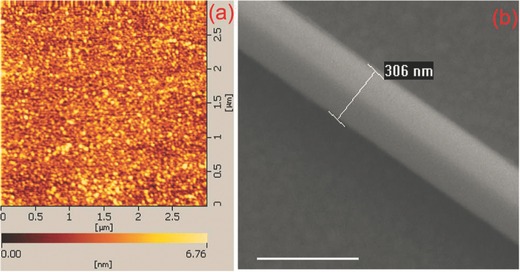
a) AFM image of Ag film, and b) a typical SEM image of Ag nanowire‐Ag film system. The scale bar in b) is 500 nm. Reproduced with permission.^89^ Copyright 2012, Royal Society of Chemistry.

**Figure 12:**
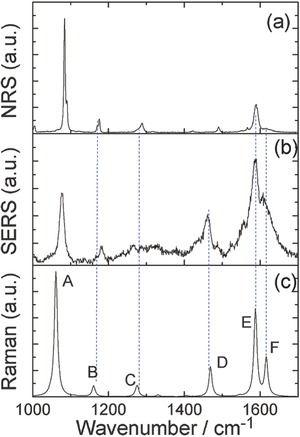
a) NRS spectrum of PATP powder, b) SERS spectrum of PATP, and c) the simulated Raman spectrum of PATP adsorbed on Ag_5_ cluster. Reproduced with permission.^89^ Copyright 2012, Royal Society of Chemistry.

**Figure 13:**
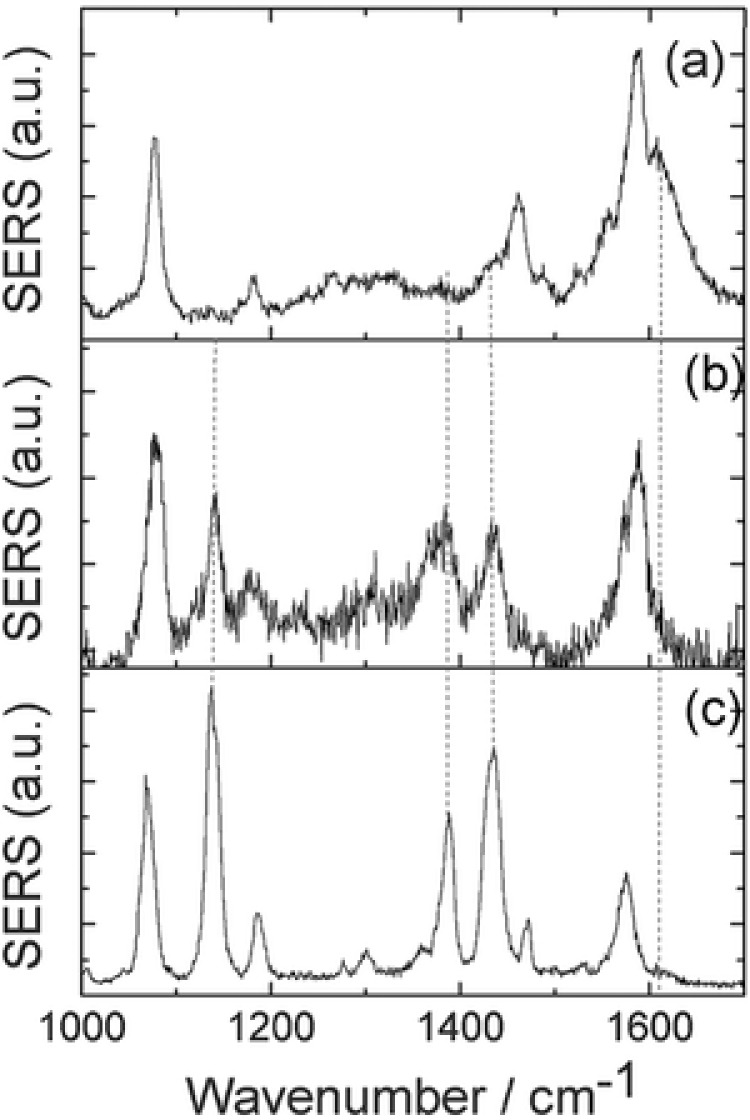
a,b) SERS spectra of PATP measured at the very beginning and after 60 s of laser radiating in Ag nanowire on Ag film system, respectively, and c) the SERS spectrum of DMAB in Ag aggregation system. Reproduced with permission.^89^ Copyright 2012, Royal Society of Chemistry.

To provide further evidence for plasmon‐driven chemical reaction of PATP being converted to DAMB, it is necessary to ascertain the real SERS spectra of PATP. By using the system of nanoparticles, the strong surface plasmonic resonance make the chemical reaction easy, and the real SERS spectra of PATP would not be discovered, which is a challenge of observation of the real SERS spectra of PATP. As shown in **Figure** **11**, by using the SERS substrate of silver nanowire on silver, film the SERS spectra of PATP was same as the normal Raman spectra of PATP, where the DMAB modes at 1140, 1390, 1432 cm^−1^ were not obtained (see **Figure** **12**), and the SERS spectra are the same as the Raman spectrum of PATP powder.^89^ Furthermore, the laser intensity dependent dynamics of surface catalytic reaction that PATP react to DMAB were also monitored (see **Figure** **13**). The reason that the genuine SERS spectrum was successfully observed is that the enhancement of electromagnetic field at the gap of silver nanowire and silver film is much weaker than that in the normal hot‐spots of silver or gold nanoparticles.^89^

Then similar experimental results have also reported by many other groups.^90^, ^91^, ^92^, ^93^ The mechanism of Plasmon‐driven chemical reaction has been also reported by Xu et al.^90^ and Tian et al.,^30^ respectively.

#### 4.2.2 Remote‐Excitation Plasmon‐Driven Chemical Reactions by Nanoparticle–Nanowire Systems

**Figure 14:**
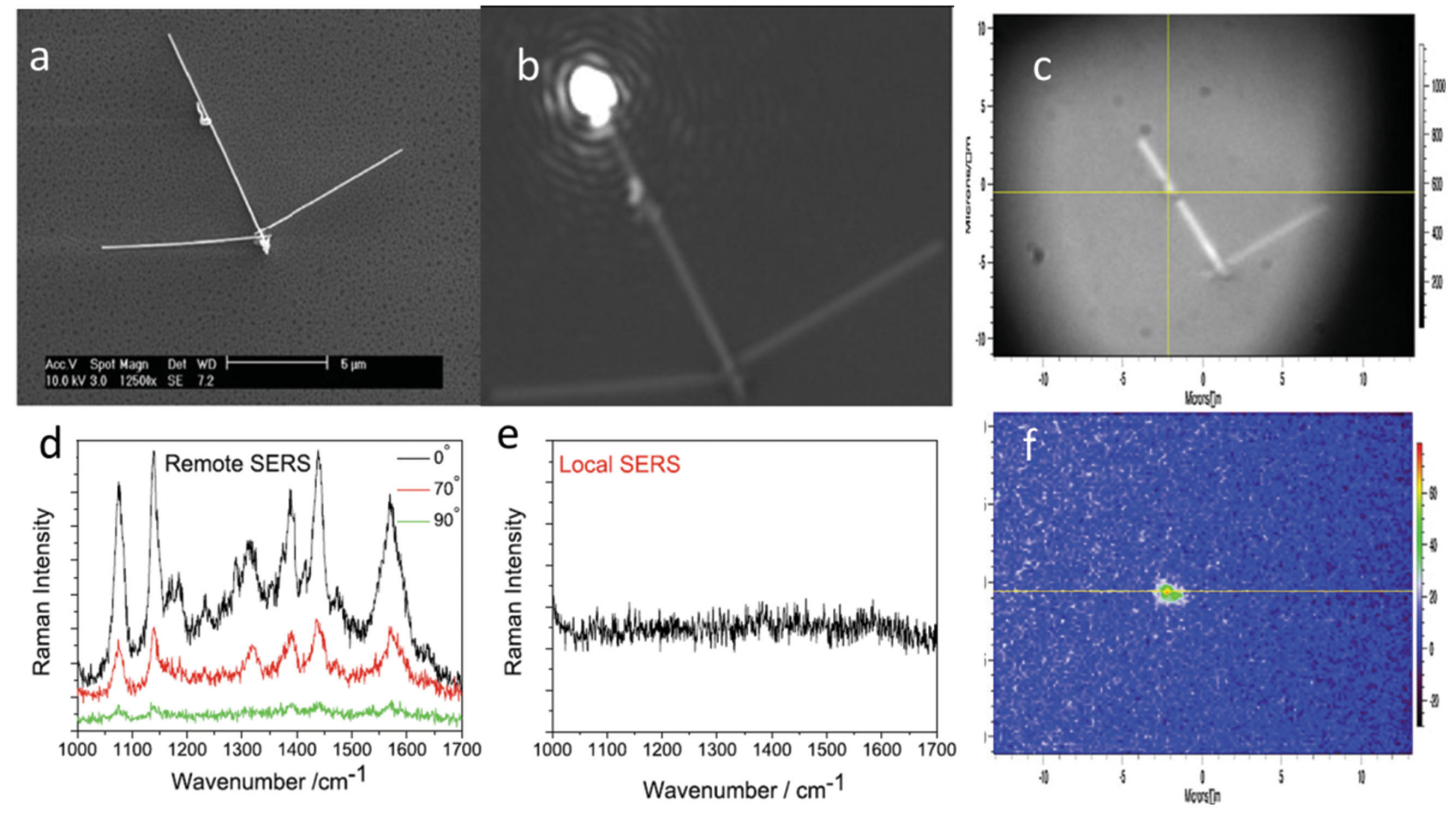
a) SEM image of Ag nanoparticles and nanowires system. b) Image of SPPs with the incident polarization along the nanowire. d) The incident polarization dependence of remote SERS spectra in this system. e) The local SERS spectrum collected at illuminating terminal. f) Raman image of Ag_17_ vibrational mode. Reproduced with permission.^53^ Copyright 2011, Springer.

Remote SERS has been introduced into plasmonic catalysis in which propagating SPPs behaved as carriers for catalysis and sensing.^53^, ^54^, ^55^ By using remote SERS technology, the plasmon catalyzed reaction of PATP to DMAB was demonstrated at the junction at the nanowire and nanoparticle system.^53^ As shown in **Figure** **14**, the SEM image and optical photo of propagating SPPs (Figure 14a,b) reveal that the distance is about 3.4 μm and the size of the excitation spot is about 130 nm. Maximal signals can be obtained when the polarization of excited laser was parallel to the nanowire waveguide. The white spot at the gap of the nanowire and nanoparticle indicates that light converted by propagating SPPs from the another side of the silver nanowire. Remote plasmon driven chemical reaction of PATP to DMAB occur by using remote SERS, and the fluctuation of polarization intensity of incident light shown by the spectra could be explained by the polarization dependence of propagating SPPs as discussed above. However, the plasmonic catalysis of PATP to DMAB cannot occur at the excited side of the nanowire, which is local SERS (Figure 14e), because the electromagnetic field intensity at the excited side is weaker than that at the hot spot of the junction, as is essential to plasmonic catalysis. The corresponding optical image and image of the Raman mode of Ag_17_ of DMAB confirm that the plasmonic catalysis occurs only at the junction of the nanowire and nanoparticle by remote SERS.

#### 4.2.3 Remote Plasmon‐Driven Chemical Reactions by Crossed‐Nanowire System

**Figure 15:**
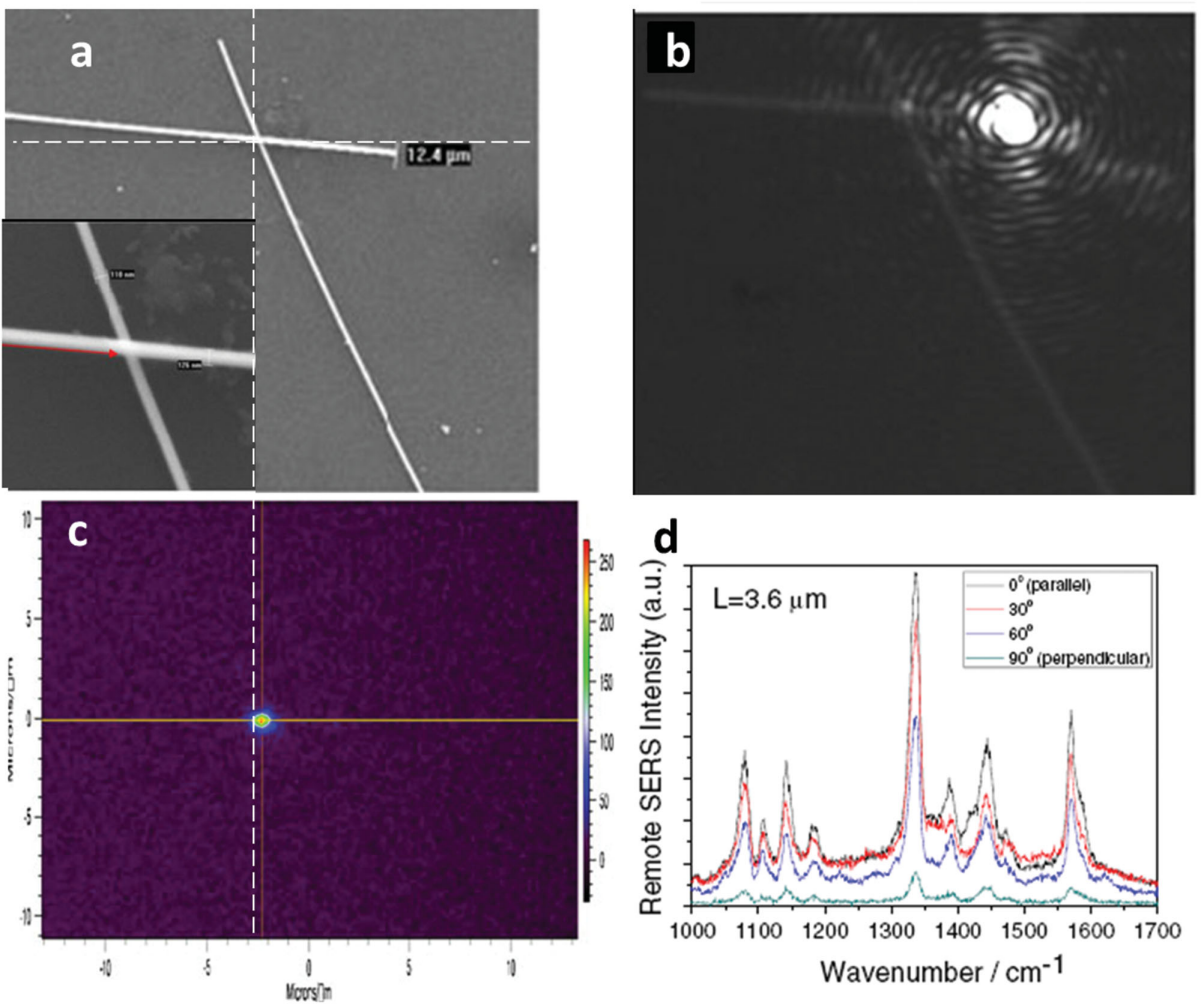
a) SEM image of silver crossed nanowires system. b) Corresponding image of SPPs of silver plasmonic waveguide. c) Ccorresponding Raman image of Ag_17_ vibrational mode. d) The polarization‐dependent remote SERS spectra of DMAB. Reproduced with permission.^54^ Copyright 2013, Springer.

In the remote SERS system of the single nanowire, the distance of remote excitation was decided by the length of nanowire. In contrast, in the nanoparticle–nanowire system, the hot spot at the nanogap of the nanoparticle and adjacent nanowire can be adjusted by changing the nanoparticle position along the nanowire. However, the distance between the nanogap and the excited side of nanowire is hard to control experimentally. A crossed nanowires system formed by two nanowires may overcome the aforementioned disadvantages. The junction of the crossed nanowires produces a hot spot that could be readily positioned through a 3D micromanipulator system under the optical microscope. We were thus able to investigate catalysis by remote SERS in crossed sliver nanowires in recent report.^54^ The remote SERS spectra at the junction indicates that the reaction of 4NBT to DMAB could be induced by propagating SPPs under a laser excitation of 632.8 nm wavelength focused at the nanowire side (**Figure** **15**). The optical image and corresponding SEM image of propagating SPPs are shown in Figure 15a,b. The magnified images show that the size of diameters of that two crossed nanowires were 126 and 110 nm, respectively. The clear white spot at the junction indicates that coupling photons emerged from propagating SPPs while the laser excited at one nanowire terminal was about 3.6 μm away. Distinct DMAB vibrational features (Figure 15d) are present in the remote SERS spectra obtained with various incident polarizations. These demonstrate that the surface catalysis reaction was remotely induced by propagating SPPs. The maximal intensity of remote SERS was observed while the polarization of incident laser was parallel to the nanowire. This result can be explained by the higher efficiency of SPPs propagation along the nanowire axis in the single‐silver‐nanowire system. To exclude the possibility that SERS signals obtained from the junction were due to background scattering or SPP propagation at the excited side, Raman images at the Ag_17_ vibrational mode of DMAB are shown in Figure 15c. The colored gap area indicates that remote plasmonic catalysis only occurred in the hot spot due to the strongly enhanced EM field. In addition, the time dependent properties of remote plasmon catalyzed reactions in such crossed nanowires system were also confirmed in this work.

#### 4.2.4 Remote Plasmon‐Driven Chemical Reactions by a Metal‐Nanowire Array‐Bundle System

**Figure 16:**
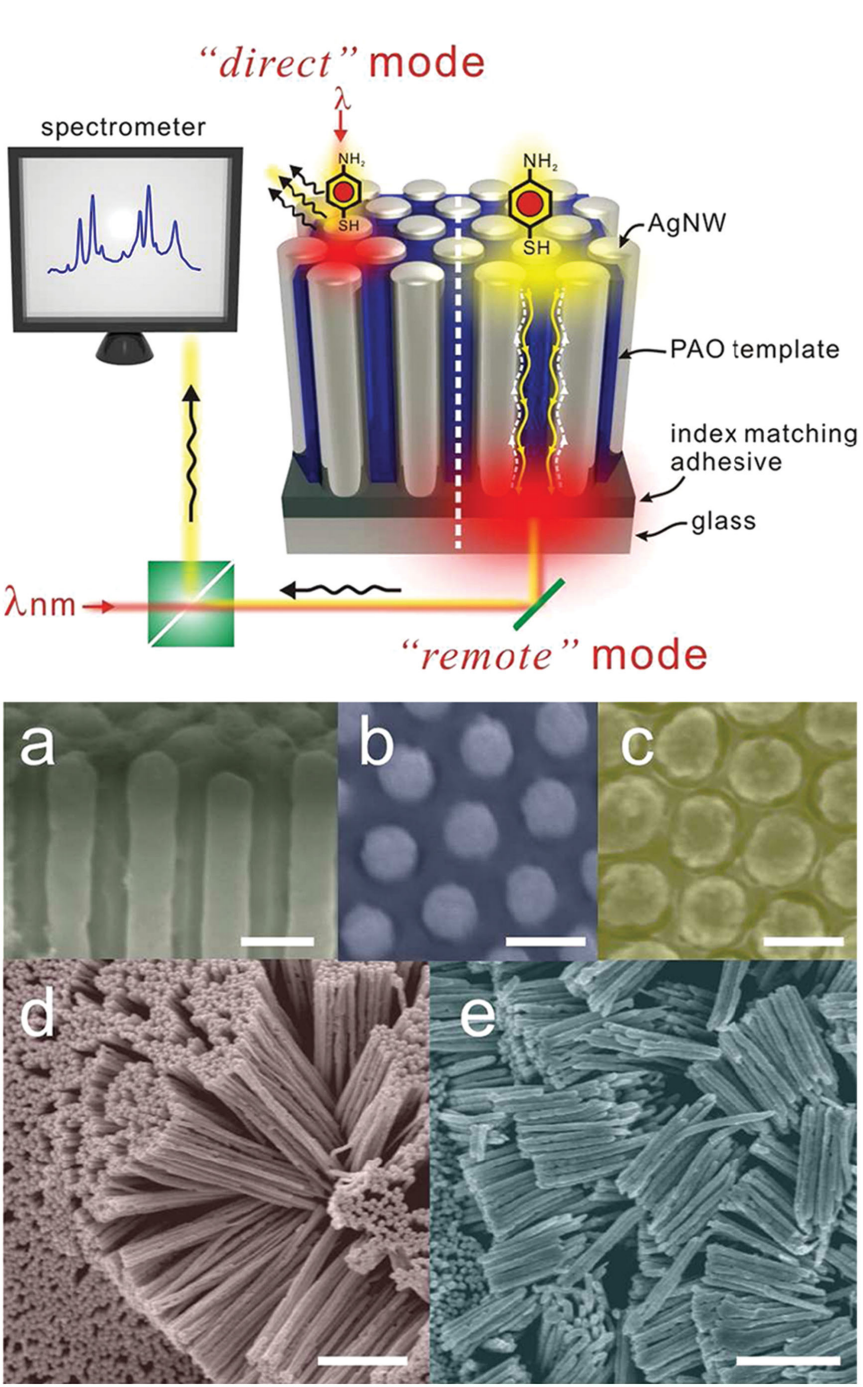
(top) The sketch of the remote and local SERS based on silver nanowire array bundles system. (bottom) SEM images of system. a) Lateral and b) top view SEM images of a silver nanowire array bundles system. c) Top view of system with 11 nm inter‐wire gaps. d,e) systems with 2.2 μm length (33 nm gap) and 1 μm (11 nm gap) after dissolving the porous alumina oxide template. Reproduced with permission.^55^ Copyright 2012, American Chemical Society.

In 2012, Moskovits' group reported another efficient remote SERS system by using nanowire bundle consists of nanowire array that produced by electrochemical deposition on a porous anodic alumina (PAA) template. Due to the strongly enhanced EM field at the nanowire gaps, this system were also used in plasmonic catalysis, such as water splitting and the reaction of PATP to DMAB.^94^, ^95^, ^96^ In this remote sensing system, the silver nanowire array was used as a probe, which can be attached to a catheter or an optical fiber to collect remote SERS signals in biological cell or liquids.^55^ The SEM images of probe structures and experiment setup are illustrated in **Figure** **16**. The anodic alumina film at top was slightly dissolved to ensure that the PATP molecules can be attached to the Ag nanowire array. An index‐matching adhesive was used to reduce the reflection from the interface between glass substrate and remote system. The bundles of silver nanowire with 11 and 33 nm gap distances are presented, while the distance between nanowires was about 100 nm. The lengths of the silver nanowire array were about 2.2 and ≈1 μm for 33 and 11 nm gap, respectively. As shown in the standard SERS, the incident laser was directed to focus on the top of the nanowires where the molecules were located. But for remote SERS, the incident laser was focused on the bottom glass substrate. In contrast to the above remote SERS systems, SPPs propagating along the silver nanowires originated not only from the incident laser, but also by the molecular Raman scattering in this nanowire array remote system. So the observation suggests that the remote distance was twice the length of nanowire array. Because of electrical resistance, SPPs propagating along the Ag nanowires could be attenuated exponentially as a function of the nanowire length, which was confirmed by the results on the remote SERS with different lengths ranging from 1 to 3 μm. The results also indicate that the Raman signals observed from larger nanowires (88 nm) were stronger than those from smaller nanowires (68 nm). The difference is due to the enhanced function of the narrower inter‐nanowire gap as a hot spot and to the lower attenuation achieved with the larger nanowire.

**Figure 17:**
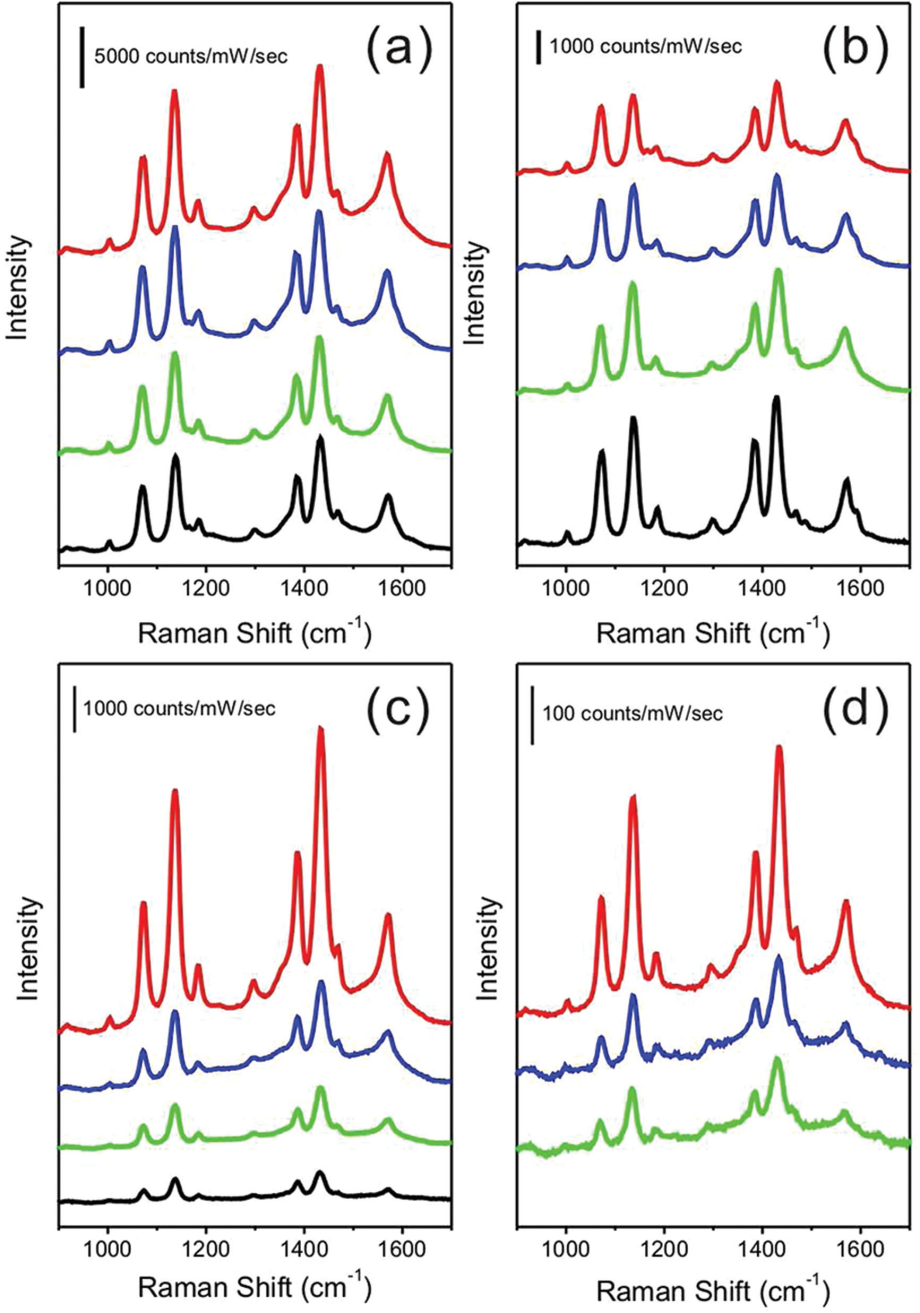
Averaged SERS spectra taken by a,b) direct excitation and c,d) remote excitation; a,c) gap of ≈11 nm with nanowire length of ≈920 nm (red), ≈1960 nm (blue), ≈2520 nm (green), and ≈3300 nm (black), respectively. b,d) Gap of ≈33 nm with NW length of ≈860 nm (red), ≈1880 nm (blue), ≈2160 nm (green), and ≈2880 nm (black), respectively. Reproduced with permission.^55^ Copyright 2012, American Chemical Society.

As shown in **Figure** **17**, excellent SERS and remote SERS spectra can be obtained by such a remote nanowire array system.^55^ Clearly, the plasmon catalyzed reaction of PATP to DMAB can be observed and monitored by remote probe, although here it was not comprehended that these SERS peaks were those of DMAB. The high remote SERS intensities indicate that the excited laser produced SPPs on the silver nanowire array can reach to the remote side of the nanowire; some energy was coupled out to create an optical near field in the gaps at the remote side where excited the molecules.

## 5 Conclusions and Perspectives

We have reviewed plasmonic waveguide for remote‐excitation surface catalytic reactions in detail, including the synthesis methods of Ag nanowires, the property and physical mechanism of plasmonic waveguides, and the successful applications of plasmonic waveguides in the field of remote‐excitation surface catalytic reactions. However, some challenges should be overcome in order to understand the full potential of remote plasmonic catalysis. More experimental and theoretical research, including on plasmon induced hot electrons, SPPs and plasmonic catalysis, is necessary in the near future so as to exploit the full potential of remote SERS systems. In addition, the distance of remote SERS based on metal nanowire waveguide is limited to a dozen μm. In the future, more methods for the synthesis of plasmonic materials should be developed, since the yield of Ag nanowire synthesized by chemical methods is limited. Stronger plasmonic waveguides should be created by nano‐antenna, since the stronger intensity of plasmonic waveguide can result in a larger probability of chemical reaction. For example, withtwo or more Ag nanowires crossed, and two or more lasers simultaneously radiated on the terminals of the nanowires, collecting the remote SERS signals at the cross point. Better substrates should be developed for decreasing the loss of plasmonic waveguide on the preparation along nanowires.
